# Inhibitory effect of IL-17 on neural stem cell proliferation and neural cell differentiation

**DOI:** 10.1186/1471-2172-14-20

**Published:** 2013-04-23

**Authors:** Zichen Li, Ke Li, Lin Zhu, Quancheng Kan, Yaping Yan, Priyanka Kumar, Hui Xu, Abdolmohamad Rostami, Guang-Xian Zhang

**Affiliations:** 1 Department of Neurology, Thomas Jefferson University, Philadelphia, PA 19107, USA; 2 Department of Pharmacy, the First Affiliated Hospital of Zhengzhou University, Henan, China

**Keywords:** NSCs, IL-17

## Abstract

**Background:**

IL-17, a Th17 cell-derived proinflammatory molecule, has been found to play an important role in the pathogenesis of autoimmune diseases, including multiple sclerosis (MS) and its animal model, experimental autoimmune encephalomyelitis (EAE). While IL-17 receptor (IL-17R) is expressed in many immune-related cells, microglia, and astrocytes, it is not known whether IL-17 exerts a direct effect on neural stem cells (NSCs) and oligodendrocytes, thus inducing inflammatory demyelination in the central nervous system.

**Methods:**

We first detected IL-17 receptor expression in NSCs with immunostaining and real time PCR. We then cultured NSCs with IL-17 and determined NSC proliferation by neurosphere formation capability and cell number count, differentiation by immunostaining neural specific markers, and apoptosis of NSCs by flow cytometry.

**Results:**

NSCs constitutively express IL-17R, and when the IL-17R signal pathway was activated by adding IL-17 to NSC culture medium, the number of NSCs was significantly reduced and their ability to form neurospheres was greatly diminished. IL-17 inhibited NSC proliferation, but did not induce cytotoxicity or apoptosis. IL-17 hampered the differentiation of NSCs into astrocytes and oligodendrocyte precursor cells (OPCs). The effects of IL-17 on NSCs can be partially blocked by p38 MAPK inhibitor.

**Conclusions:**

IL-17 blocks proliferation of NSCs, resulting in significantly reduced numbers of astrocytes and OPCs. Thus, in addition to its proinflammatory role in the immune system, IL-17 may also play a direct role in blocking remyelination and neural repair in the CNS.

## Background

Due to the capability of NSCs to undergo self-renewal and to differentiate into multiple cell types, NSC-based transplantation has become a potential therapeutic approach in the treatment of neurological disorders such as multiple sclerosis (MS) [1,2]. The majority of NSCs come from two areas: the subventricular zone (SVZ) and the subgranular zone of the hippocampus [3,4]. Recent reports suggest that, under normal conditions, proliferation and differentiation of NSCs are necessary for neural repair [5]. However, this function is dramatically reduced in MS, resulting in the breakdown of spontaneous remyelination and neural recovery [6].

Interleukin-17 (IL-17), an inflammatory cytokine generated by Th17 cells, has been implicated in the development of MS and its animal model, experimental autoimmune encephalomyelitis (EAE) [7,8]. By increasing production of several chemokines and cytokines in the central nervous system (CNS) and modulating the inflammatory response, the IL-17/IL-17R pathway plays a critical role in the development of MS [9,10]. Although EAE and MS have been considered typical Th1 cell-mediated diseases, growing evidence suggests that Th17 cells play an important role in the effector mechanisms of these, and other, autoimmune diseases [9,11-13]. Thus, any factor that directly impairs development of Th17 cells, or a deficiency in factors that promote this lineage (IL-1, IL-6, IL-23), consistently abrogates EAE [14,15]. IL-17 is the hallmark cytokine of Th17 lineage, which binds to the heteromeric transmembrane receptor, resulting in recruitment of Act1 and formation of a signaling complex that facilitates inflammatory responses [16]. Mice that lack the IL-17 or IL-17 receptor are less susceptible to EAE induction, and IL-17-specific inhibition attenuates inflammation, suggesting that IL-17 signaling plays a critical role in the effector stage of EAE [17]. Importantly, increased IL-17 production and mRNA expression have been reported in MS patients with active disease [18,19].

However, to date, there have been no reports about the action of IL-17 on NSCs, or on the question whether NSCs express IL-17 receptors. In the current study, we thus address this important question, and investigate the effect of IL-17 on NSC proliferation, differentiation and cell death.

## Methods

### NSC cultures and differentiation

Mouse NSCs were generated from 14-day-old embryos (E14) from C57Bl/6 mice. Briefly, whole brains of C57BL/6 E14 mouse embryos were harvested under sterile conditions and placed in DMEM medium. After a brief washing with DMEM medium, tissues were cut into 1 mm^3^ pieces and suspended in 2 ml 0.25% trypsin with EDTA (Invitrogen, NY, USA), mechanically dissociated for 2 min and incubated at 37°C for 30 min. After filtration through a 70 μm cell strainer (BD Bioscience, San Jose, CA), the cell suspension was washed twice with 10 ml DMEM medium. Cells were resuspended in serum-free DMEM/F-12 (Invitrogen, NY, USA) supplied with 2% B27 supplements (Invitrogen, NY, USA), 20 ng/ml epidermal growth factor (EGF, Peprotech, Rocky Hill, NJ, USA) and 20 ng/ml basic fibroblast growth factor (b-FGF, Peprotech, Rocky Hill, NJ), along with 100 IU/ml penicillin and 100 μg/ml streptomycin (Sigma-Aldrich, MI, USA). Cells were then transferred to poly-L-lysine coated 6-well plates (BD Bioscience, San Jose, CA) at a density of 2 × 10^5^ cells/ml and maintained in culture at 37°C. Culture medium was changed every 3 days. Neurospheres were formed after 3-4 days of culture. For passaging, free-floating neurospheres were collected and dissociated with Accutase cell detachment solution (Innovative Cell Technologies, San Diego, CA) into small neurospheres or single cells and re-seeded at a density of 1 × 10^5^ cells/ml in the same medium. NSCs at passage 4-15 were used in all *in vitro* experiments. To induce NSC differentiation, dissociated single cells or small neurospheres were incubated in stem cell differentiation medium (NSC basal medium plus 10% NSC differentiation supplements, Stemcell Technologies) for 7 to 14 days and processed for immunofluorescence. All animal protocols were approved by the Institutional Animal Care and Use Committee of Thomas Jefferson University, following NIH guidelines.

### IL-17R immunostaining

Cells grown on coated cover slips for two days and fixed with 4% paraformaldehyde in PBS (Cellgro Mediatech, USA) were blocked in PBS/0.1% saponin/5% goat serum and incubated with primary antibody at 4°C overnight. Rabbit anti-IL-17R antibody (Santa Cruz, CA, USA) was used to determine IL-17 receptor. Briefly, cells were cultured on slides with stem cell medium, then were washed 2 times with PBS for 5 min, fixed in 3.7% PFA for 10 min at room temperature and washed 3 times with PBS. Blocking was performed in 10% of appropriate serum for 2 hours at room temperature. Cells were then incubated with anti-IL-17R (1:100) overnight at 4°C. After being washed twice in PBS with 0.5% Triton X-100, secondary antibodies were applied for 1 h at room temperature. Cells were then washed, mounted onto Mowiol, and visualized by fluorescence microscopy (Olympus I X-80) with a 20 PlanApo oil immersion objective (1.0 numerical aperture). For visualizing all cells, nuclei were counterstained with DAPI. In this experiment omitting primary antibody was used as control. Images were acquired with a SensiCamQE High Performance CCD Camera.

### Real-time PCR

Total RNA was isolated from NSCs in the same culture conditions as those used in immunostaining. RNAs isolated from primary oligodendrocytes (>93% GalC^+^) of wild type B6 mice served as IL-17R positive control [20], and of IL-17R-deficient mice (the Jackson Laboratory) as negative control. For quantitative real-time PCR of IL-17R, specific primers were generated as follows: IL-17RrealF: 5′-AGGTCCAGCCCTTCTTCAGCA-3′, IL-17RrealR: 5′-GCTTGGGAACTGTGGTATTTGA- -GATTA-3′. High Capacity cDNA Reverse Transcription Kit (Invitrogen), RNeasy Mini Kit and QuantiFast SYBR Green PCR Kit (QIAGEN) were used for real-time PCR according to the manufacturer’s instructions.

### Analysis of neurosphere growth

To determine the neurosphere volume of stimulated NSCs, these cells were cultured at 200 cells/ml in 96-well plates. These cells were cultured in the presence of IL-17 at different concentrations (0, 5, 10, 25, 50, 100 ng/ml) for 96 hours. NSCs in separate wells were cultured in the presence of TNF-α at 25 ng/ml as positive control, given its cytotoxicity to neural cells [20], while IL-10 at 50 ng/ml was also used as control, which does not interfere NSC proliferation [21]. The number of living neurospheres was counted under inverse microscope (ECLIPSE TS-100, Nikon, Japan).

### Analysis of cell numbers

To determine the actual number of cells in neurospheres, we did a cell number count after dissociating neurospheres into single cells. Briefly, NSCs were cultured at 1.5 × 10^5^ cells/ml in 24 well plates. These cells were cultured in the presence of IL-17 at different concentrations (0, 5, 10, 25, 50, 100 ng/ml) for 96 hours, and in the presence of IL-10 at 50 ng/ml [21] or TNF-α at 25 ng/ml [20] as control. Neurospheres were collected, dissociated and cell number was counted under inverse microscope (ECLIPSE TS-100, Nikon, Japan).

### MAPK signaling pathway analysis

We then determined p38 MAPK signaling activation in NSCs by western blot. Briefly, cells were cultured at 2 × 10^5^ cells/ml in 6-well plates for 2 days. Two wells were added with IL-17; 2 wells were added with IL-17 and inhibitor of p38 MAPK (SB203580) (Cell Signaling) at 15 mM. Then cells were lysed in lysis buffer (Cell Signaling) supplemented with protease/phosphatase inhibitor cocktail (Cell Signaling). Cell lysates were separated by 12% Tris-Glycine Gels (Novex® 12% Tris-Glycine Mini Gels 1.0 mm, 12-well) and transferred onto Immun-Blot PVDF membrane (Bio-Rad Laboratories). Membranes were blotted with primary antibodies followed by incubation with HRP-conjugated secondary antibodies. The blots were developed by ECL reagents and exposed on HyperFilmTM (Amersham). The following antibodies were used for western blotting: p38 MAPK (D13E1) XP® Rabbit mAb (Cell Signaling), β-Actin (C-4) (Santa Cruz Biotechnology); anti-rabbit IgG HRP-linked antibody (Cell Signaling) and goat anti-mouse IgG-HRP (Santa Cruz Biotechnology).

### Analysis of cell death and apoptosis

After incubation at room temperature for 5 min in the dark, cells were trypsinized and resuspended in 50 μL staining buffer containing 100 ng propidium iodide (PI) (Sigma-Aldrich, MI, USA), and then analyzed by flow cytometry. Dead cells were defined as PI positive. Percentages of PI positive cells among cells were calculated.

To assess apoptosis of stimulated cells, NSCs were cultured at 2 × 10^5^ cells/ml in 96 well plates and stimulated with IL-17 (25 ng/ml) for 48 hours. Cells were harvested, then stained with Annexin-V (BD Biosciences, CA, USA), and PI (Sigma-Aldrich, MI, USA) according to the manufacturer’s instructions. Briefly, cells were washed twice with PBS and then resuspended in binding buffer. 5 μl Annexin-V was added to the 100 μl solution in a tube and incubated for 15 min at room temperature in the dark. Cell apoptosis was analyzed by flow cytometry within 1 hour. Apoptotic cells were defined as Annexin-V positive.

### Analysis of cytotoxicity

To determine the cytotoxicity of IL-17 on stimulated NSCs, extracellular LDH activity was detected with an LDH Cytotoxicity Detection Kit (Clontech Laboratories, Mountain View, CA) following the manufacturer’s instructions. Briefly, NSCs were cultured at 1 × 10^5^ cells/ml in 96-well plates. These cells were cultured in the presence of IL-17 at different concentrations (0, 5, 10, 25, 50, 100 ng/ml) for 48 hours. The plates were then centrifuged at 250 g for 10 min., and 100 μl of supernatant from each well was transferred into the corresponding well of a 96-well flat-bottom plate. 0.1 ml of freshly prepared Reaction Mixture was added to each well and incubated for up to 30 min at room temperature, protected from light. Absorbance of the samples was measured and the cytotoxicity percentage was calculated according to the manufacturer’s instructions.

### Proliferation assay

[^3^H]-thymidine DNA incorporation was measured in stimulated cells. Briefly, NSCs were cultured at 1.5 × 10^4^ cells/ml in 96-well plates. These cells were cultured in the presence of IL-17 at different concentrations (0, 5, 10, 25, 50, 100 ng/ml) for 48 hours. 1 μCi/well 3H-Thymidine was added to each well. Plates were incubated for 18 hours. 30 μl NaOH 1N solution (Sigma Diagnostics, MO) was added to each well to lyse the cells; then 30 μl hydrochloric acid 1N solution was added to each well to neutralize NaOH (Fisher Scientific, NJ). After supernatant had been thoroughly mixed, radioactivity was measured in a beta-counter.

### Immunostaining

Cells grown on coated coverslips and fixed with 4% paraformaldehyde in PBS (Cellgro Mediatech, USA) were blocked in PBS/0.1% saponin/5% goat serum and incubated with primary Abs at 4°C overnight. β-III-tubulin was used as neuron marker; GFAP as an intracellular astrocytic marker; NG2 as oligodendrocyte progenitor cell (OPC) marker and Sox2 as NSC marker. Briefly, cells were cultured on slides with stem cell medium, washed 2 times with PBS for 5 min, fixed in 3.7% PFA for 10 min at room temperature and washed 3 times with PBS. Blocking was performed in 10% appropriate serum for 2 hours at room temperature. Cells were then incubated with anti-β-III-tubulin (1:150); anti-GFAP (1:150); anti-NG2 (1:150) and anti-SOX2 (1:100) overnight at 4°C. After washing twice with PBS containing 0.5% Triton X-100, secondary antibodies were applied for 1 h at room temperature. Cells were then washed, mounted onto Mowiol, and visualized by fluorescence microscopy (Olympus I X-80) with a 20 PlanApo oil immersion objective (1.0 numerical aperture). For visualizing all cells, the nuclei were counterstained with DAPI. In this experiment omitting the primary antibody was used as control. Images were acquired with a SensiCamQE High Performance CCD Camera.

### Statistical analysis

Data are presented as the mean ± SE of 3–6 independent experiments, each carried out in triplicate or quadruplicate. Comparisons were analyzed using one-way analysis of variance (ANOVA), followed by a post-hoc Bonferroni's multiple comparison test. Statistical significance was established at p<0.05. The tables and graphs of the original data were produced using GraphPad Prism software version 5.00 for Windows (GraphPad, USA).

## Results

### NSCs express IL-17R

**Figure 1:**
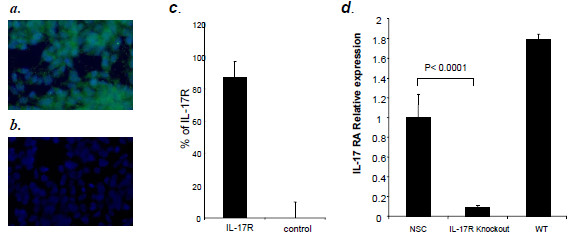
**IL-17R expresses in NSCs. **Dissociated single cells were incubated in stem cell culture or differentiation medium in 12-well plates for 2 days and processed for immunofluorescence. (**a**) Immunocytochemistry images of neural stem cells cultures labeled for IL-17R (green) and counterstained with the nuclei dye DAPI (blue). (**b**) IL-17R antibody was omitted to serve as control. Magnification ×20 for A, ×20 for B. (**c**) Plot depicts the percentage of IL-17R positive cells. (**d**) RT-PCR for IL-17R expression in NSCs. Primary oligodendrocytes (>93% GalC^+^) of wild type B6 mice served as positive control and of IL-17R knockout mice as negative control. Mean percentages were determined from five independent experiments. WT: wild type.

Before investigating the direct effect of IL-17 on NSCs, we measured the expression level of IL-17R in NSCs. With immunofluorescence, we found that IL-17R is expressed in *in vitro* cultured NSCs (Figure 1a, b and c). IL-17R expression on NSCs was further confirmed by RT-PCR, with spinal cord tissues of wild type mice as positive control [20] and of IL-17R knockout mice as negative control (Figure 1d). These findings demonstrate that IL-17R is expressed constitutively in NSCs, indicating that IL-17 can exert a direct effect on these cells.

### IL-17 can reduced neurosphere formation and inhibit NSC number increase

**Figure 2:**
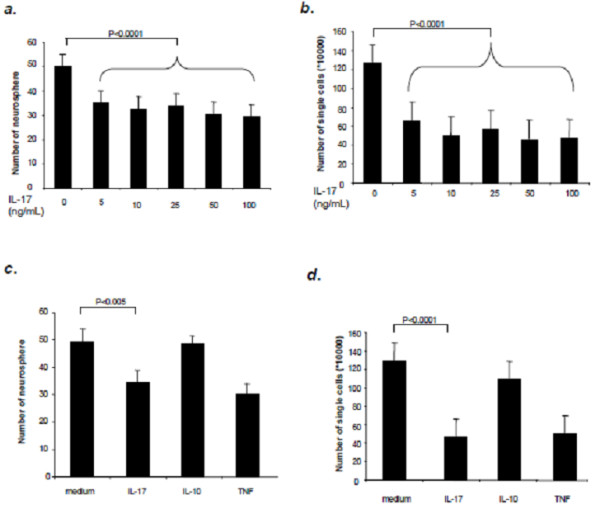
**IL-17 induces a decrease in both neurosphere volume and cell number. **NSCs were cultured at 400 cells/ml in 96- or 12-well plates and treated with IL-17, TNF-α and IL-10 for 96 h, as described in Materials and Methods. (**a**) NSCs treated with different concentrations of IL-17; (**b**) Number of NSCs treated with different concentrations of IL-17; (**c**) NSCs treated with IL-17 (25 ng/ml), TNF-α (25 ng/ml) and IL-10 (50 ng/ml) alone; (**d**) Number of NSCs treated with IL-17 (25 ng/ml),TNF-α (25 ng/ml) and IL-10 (50 ng/ml) alone. Cell number and the number of living neurospheres were measured using an inverse microscope. Mean percentages were determined from five independent experiments. NS, not significant.

To investigate the effects of IL-17 on neurosphere formation and cell number changes in NSCs, we compared NSC in culture medium with IL-17. Single NSCs from passage 10 onwards were cultured in 96 well plates. After 4 days’ culture with IL-17 at different concentrations (0, 5, 10, 25, 50, 100 ng/ml), neurosphere volumes were then measured. We found that IL-17 can significantly reduce the number of newly formed neurospheres in culture medium (Figure 2a), while there was no difference in the formation of NSC neurospheres at various IL-17 concentrations. We therefore analyzed the actual cell number change after stimulation. As expected, IL-17 significantly decreased the cell number in the treated group compared with the medium control (Figure 2b). Similar results were obtained when TNF-α was added as a positive control, while there was no influence when IL-10 was added as a negative control (Figure 2c and 2d), consistent with previous observation [21].

### IL-17 does not induce NSC apoptosis

**Figure 3:**
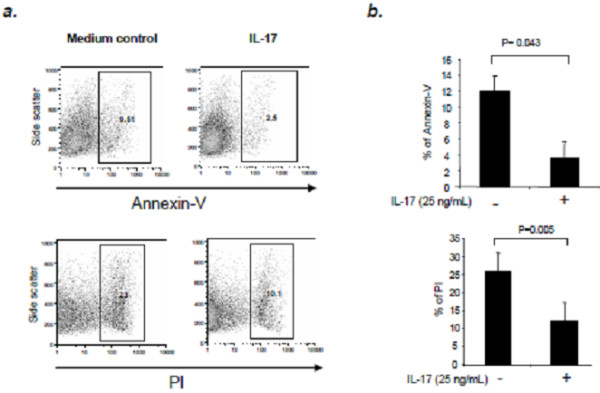
**IL-17 does not induce Annexin-V or PI uptake.** NSCs cells were cultured in 6-well plates and treated with IL-17 (25 ng/ml) for 48 h, followed by incubation with Annexin-V dye and PI and FACS analysis. (**a**) Representative histograms demonstrate the distribution of Annexin-V positive and PI positive cells in treated/untreated NSCs. (**b**) Plot depicts the percentage of Annexin-V positive and PI positive cells in treated/untreated NSCs. Data represent mean ± SE in duplicate samples from three identical experiments. NS, not significant.

We investigated whether IL-17 would induce apoptosis of NSCs. As shown in Figure 3, Annexin-V positive cells were significantly decreased in NSCs upon treatment with IL-17 compared with the control group. At the same time, FACS analysis revealed a decrease in the percentage of PI positive NSCs (Figure 3) upon treatment with IL-17 compared with controls. Our data provide evidence that the inhibitory role of IL-17 in NSC proliferation does not result from cell apoptosis.

### IL-17 does not enhance cytotoxicity in NSCs

**Figure 4:**
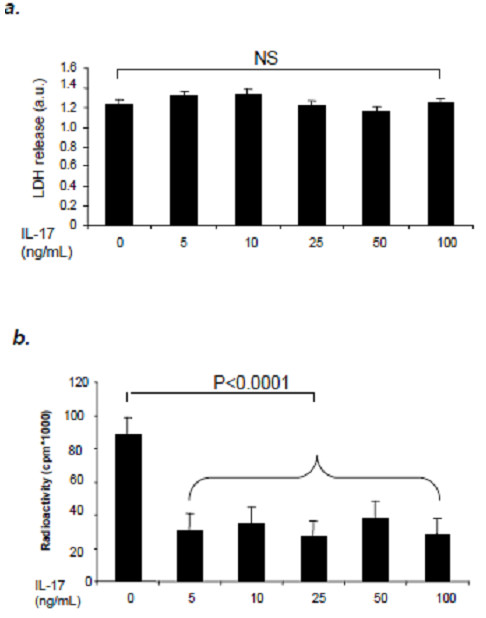
**IL-17 does not induce cell death in NSCs, but decreases NSC proliferation. ** Cells were cultured in 96-well plates and treated with IL-17 for 48 h as described in Materials and Methods. (**a**) Plots depict LDH release in NSCs treated with different concentrations of IL-17; (**b**) Plots depict radioactivity in NSCs treated with different concentrations of IL-17. Data represent mean ± SE in triplicate samples from four independent experiments. NS, not significant.

Cytotoxicity is one of the major causes of decrease in cell numbers. To investigate whether IL-17 has cytotoxicity to NSCs when inhibiting NSC proliferation, LDH release from NSCs was detected after treatment with IL-17 for 2 days. According to the manufacturer’s instructions, we mixed medium with Reaction Mixture and measured the absorbance of the samples. Our data showed that, whether or not NSCs were treated with IL-17, there was no significant change in LDH release from NSCs (Figure 4a). Our data demonstrate that IL-17 shows no cytotoxicity in NSCs.

### IL-17 inhibits NSC proliferation, partially through activating p38 MAPK pathway

Having shown that the reduction of NSC number was not due to IL-17-induced cell killing, we next explored whether this reduction resulted in an inhibitory effect of IL-17 on NSC proliferation. We used ^3^H-TdR incorporation to measure proliferation and found that adding IL-17 to culture significantly inhibited proliferation of cultured NSCs (P<0.001; Figure 4b). These results indicate that IL-17 plays an inhibitory role in NSC proliferation.

**Figure 5:**
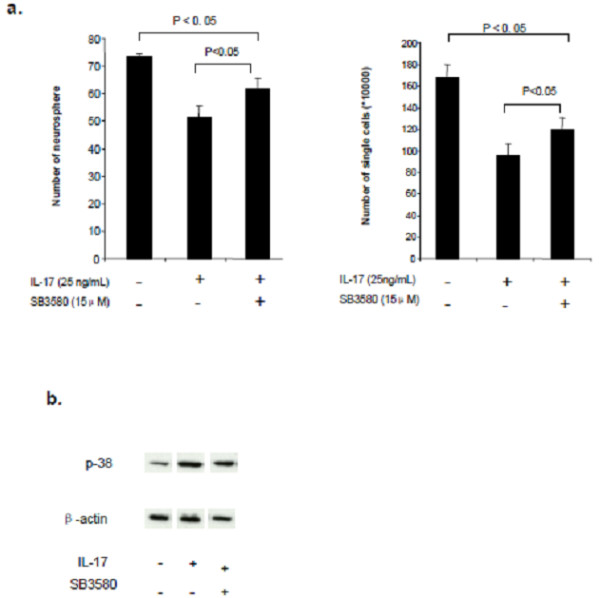
**IL-17 increases p38 MAPK level in NSCs. **NSCs were cultured at 400 cells/ml in 6-well plates and treated with IL-17 for 48 h, as described in Materials and Methods. SB203580 was added to culture medium 2 h before IL-17 was added. (**a**) Newly formed NSCs treated with IL-17 (25 ng/ml), with or without addition of SB203580 (15 μM). (**b**) Number of NSCs treated with IL-17 (25 ng/ml), with or without addition of SB203580 (15 μM). (**c**) Western blot for p38 MAPK level in NSCs. Mean percentages were determined from three independent experiments. NS, not significant.

We then explored the downstream signal pathway for IL-17 induced proliferation decrease. To determine whether activation of p38 MAPK is involved in IL-17 stimulation in NSCs, we used SB203580 (p38 inhibitor) to block p38 MAPK. Our data show that decrease in newly formed neurosphere and total cell number after IL-17 treatment is inhibited by adding SB203580 (15 μM) to culture medium (Figure 5a). The induction of p38 MAPK by IL-17 and inhibition by SB203580 in NSCs were confirmed by western blot (Figure 5b).

### IL-17 interferes with NSC differentiation

**Figure 6:**
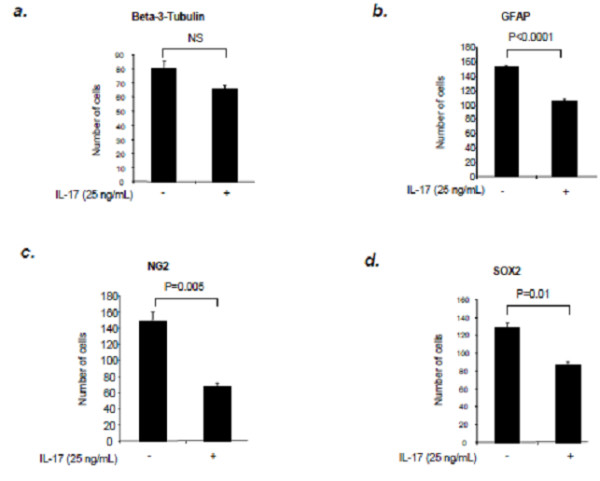
**IL-17 interferes with NSCs differentiation.** Dissociated single cells were incubated in stem cell differentiation medium in 12-well plates and treated with IL-17 (25 ng/ml) for 14 days, then were processed for immunofluorescence. IL-17 treatment did not significantly change the number of β-III-tubulin positive cells (**a**), but significantly decreased the numbers of GFAP positive cells (**b**), NG2 positive cells (**c**) and SOX2 positive cells (**d**). Data represent mean ± SE in duplicate samples from three identical experiments. NS, not significant.

A crucial property of NSCs is the capacity to differentiate into more specialized cells. To study the role of IL-17 activation in NSC differentiation, we cultured NSCs on a poly-L-lysine pre-coated micro glass cover in 12-well culture plates with differentiation culture medium for 14 days in order to induce differentiation of NSCs [1]. Cultured cells were treated with IL-17. We used the following neural specific markers: β-III-tubulin (neurons), GFAP (astrocytes), NG2 (OPCs) and SOX2 (undifferentiated NSCs). As shown in Figure 6, while the number of β-III-tubulin positive neurons was not significantly reduced, adding IL-17 to NSC culture resulted in a decrease in numbers of GFAP^+^ astrocytes, NG2^+^ OPCs and SOX2^+^ undifferentiated NSCs. These results indicate that IL-17 stimulation negatively influences NSC differentiation into later stages of neural cells.

## Discussion

NSCs are a unique population of cells that exhibit stem cell properties, including self-renewal (production of a large number of progeny) and multipotency (differentiation of the progeny into the three primary CNS phenotypes: neurons, astrocytes and oligodendrocytes) [22]. NSCs that have been isolated from adult brain can be maintained *in vitro* for extended periods of time without losing their proliferation or differentiation potential [22,23]. Because NSCs have the ability to support neurogenesis within restricted areas throughout adulthood and can undergo extensive *in vitro* expansion, they have been proposed as a renewable source of neural precursors for regenerative transplantation in various CNS diseases, including degenerative disorders, injury and cancer [24,25] and EAE [21,26,27]. These cells reached multiple demyelinating areas of the CNS and ameliorated EAE clinically and pathologically to a similar extent when injected either intravenously or intraventricularly [27]. However, in inflamed foci, such as occur in MS and EAE, which are a hostile microenvironment for NSCs, the migration and proliferation of NSCs are inhibited, resulting in a failure of spontaneous remyelination and neural repair [28]. It is thus important to identify immune cells and/or proinflammatory mediators that are responsible for this pathogenic outcome.

IL-17A, which has been called IL-17, is the prototypical cytokine of the IL-17 family, which comprises IL-17A-F [16]. IL-17A and IL-17F trigger signaling via their receptor, a heterodimeric molecule composed of IL-17RA and IL-17RC [29]. IL-17 activates NFκB signaling and induces several proinflammatory cytokines and chemokines, in particular, CCL20, which can attract CCR6-expressing Th17 cells [30]. In the CNS, IL-17 activates microglial cells [31] and induces oligodendrocyte cell death [20]; it is thus pathogenic in CNS inflammatory demyelination. However, the role of IL-17 signaling in NSCs is not yet known. This question is important given the critical role of these cells in remyelination and neural repair following brain damage. The capacity of NSCs for self-renewal and for generating functional differentiated cells makes them an attractive potential therapeutic tool for the treatment of neurological disorders. Indeed, NSCs have recently emerged as a potential therapeutic approach in the treatment of MS [2]. In the present study, we demonstrate that IL-17 significantly reduces NSC number by inhibiting the proliferation of these cells, and is thus a novel mechanism underlying the pathogenesis of Th17 cells in the development of CNS inflammatory demyelinating diseases such as MS.

Previous studies have shown that IL-17/IL-17R interactions use TRAF6 to transduce its signal in immune cells [32], and is a well-studied multi-pathway process of cell death in neural cells [33]. At the same time, IL-17 mediated activation of JNK1/2 is reported to be involved in cell death [34,35]. Moreover, IL-17R has been found to express in oligodendrocytes, the myelinating cells, and adding IL-17 to culture induces significant oligodendrocyte apoptosis [20]. We hypothesized that NSC apoptosis would be increased after treatment with IL-17. To this end, we tested whether IL-17 had induced NSC apoptosis. Surprisingly, our findings showed that, compared with control, apoptosis in treated NSCs had decreased. Further, we also investigated whether this proinflammatory mediator induced cytotoxicity of these cells, which is an important pathway involved in cell death [36,37]. Similarly, IL-17 did not induce lactate dehydrogenase (LDH) release, which is a reliable and simple approach for defining cell death [38]. Together, these results indicate that the decrease in cell number in treated NSCs resulted from cell proliferation inhibition, not cell death.

The mitogen activated protein kinase (MAPK) family is composed of three main members, including JNK, ERK and p38 MAPK, which can translocate from cytoplasm to nucleus, and induce a series of inflammatory actions in cells [39]. Among them, the interaction between p38 MAPK and IL-17 is well known for its important role in immunity. For example, IL-17 stimulation induced a high level of proinflammatory cytokine production via the Erk1/2, p38 MAPK, PI3K/Akt, and NF-κB pathways [40]. On the other hand, activation of p38 MAPK signaling pathway is essential for in vitro and in vivo IL-17 production, and is required for the development and progression of CNS proinflammatory demyelination [41]. p38 MAPK signaling has also been found to be involved in insufficient NSC proliferation; targeting p38 restored these cells and corrected neuromotor deficits in an ataxia-telangiectasia mouse model [42]. The importance of p38 MAPK activation in the inhibition of NSC proliferation has also been observed by culturing NSCs with IL-1β, another proinflammatory cytokine that is involved in CNS proinflammatory demyelination [43]. We demonstrated that the p38 MAPK level in NSCs increased after IL-17 stimulation and that p38 MAPK inhibitor can release the anti-proliferation effect of IL-17. We also showed that the inhibition of NSC proliferation by IL-17 can be partially reversed by p38 MAPK inhibitor. Thus, in addition to its proinflammatory role in the immune system, IL-17 also play a direct role in blocking myelination and neural repair in EAE/MS though the MAPK pathway.

The capacity of NSCs for neural cell differentiation makes these cells a promising potential therapeutic tool for the treatment of nervous system disorders [1,2]. Increasing evidence suggests that NSCs go through a process of self-renewal, proliferating and differentiating into the appropriate lineage when inflammatory damage or injury occurs in the nervous system [44]. To determine the functions of IL-17 on NSC differentiation, we investigated whether IL-17 might hamper the capacity of NSCs to differentiate into neurons, astrocytes and OPCs. While IL-17 stimulation drove NSCs to differentiate into a smaller number of neurons, astrocytes, it resulted in significantly lower numbers of NSCs differentiating into astrocytes and OPCs, cells crucial for remyelination [21], as well as undifferentiated NSCs. Given that OPCs are the precursor cells of oligodendrocytes, the only myelinating cells in the CNS, reduced OPC differentiation from NSCs could be a potential mechanism of IL-17 pathogenesis in the failure of remyelination in MS/EAE [45]. Further, given that astrocytes play an important role in the production of neurotrophic factors and support neural repair [46], reduction of these cells by IL-17 would result in incomplete neural repair after CNS damage. These results, combined with the proinflammatory effect of IL-17 in the peripheral immune system and in the CNS, represent a novel mechanism underlying the failure of spontaneous remyelination and neural repair in the pathogenesis of MS.

## Conclusions

We have demonstrated that NSCs constitutively express IL-17R, and that IL-17 significantly reduces neurosphere formation, absolute number and cell proliferation of NSCs, and their differentiation into neural cells. Thus, in addition to its proinflammatory role in the immune system, IL-17 may also play a direct role in inducing demyelination and blocking neuronal repair in EAE/MS.

## Competing interests

The authors declare no competing interests.

## Authors’ contributions

The authors have made the following declaration about their contributions: ZL, LZ, GXZ conceived and designed the experiments. ZL, KL performed the experiments. ZL, KL, LZ, QK, YY, HX, AMR, GXZ analyzed the data and wrote the paper. All authors read and approved the final manuscript.
